# Association of miR‐499, miR‐27a, and miR‐34a Gene Polymorphisms With Susceptibility to Type 2 Diabetes Mellitus in an Iranian Population: A Case–Control Study

**DOI:** 10.1155/bmri/8927160

**Published:** 2026-09-09

**Authors:** Mehrdad Behdani, Mohammad Hashemi, Mahmoud Ali Kaykhaei, Masoumeh Bahari, Mohsen Taheri, Gholamreza Bahari

**Affiliations:** ^1^ Department of Clinical Biochemistry, School of Medicine, Zahedan University of Medical Sciences, Zahedan, Iran, zaums.ac.ir; ^2^ Department of Endocrinology, School of Medicine, Zahedan University of Medical Sciences, Zahedan, Iran, zaums.ac.ir; ^3^ Department of Chemistry, Faculty of Sciences, University of Sistan and Baluchestan, Zahedan, Iran, usb.ac.ir; ^4^ Genetics of Non-Communicable Disease Research Center, Zahedan University of Medical Sciences, Zahedan, Iran, zaums.ac.ir; ^5^ Cellular and Molecular Research Center, Resistant Tuberculosis Institute, Zahedan University of Medical Sciences, Zahedan, Iran, zaums.ac.ir

**Keywords:** gene polymorphism, genetic susceptibility, microRNA, Type 2 diabetes mellitus

## Abstract

**Background:**

Type 2 diabetes mellitus (T2DM) is a multifactorial metabolic disorder influenced by both environmental and genetic factors. MicroRNAs (miRNAs) are involved in the regulation of insulin signaling, inflammation, and pancreatic *β*‐cell function. Genetic polymorphisms in miRNA‐related regions may contribute to interindividual differences in T2DM susceptibility, although their functional effects remain uncertain.

**Objective:**

This study is aimed at evaluating the association of five miRNA polymorphisms, miR‐499 rs3746444, miR‐27a rs895819/rs895519, miR‐34a rs6577555, miR‐124a rs531564, and miR‐146a rs2910164, with susceptibility to T2DM in an Iranian population.

**Methods:**

This case–control study included 200 patients with T2DM and 202 unrelated healthy controls from southeastern Iran. Genotyping was performed using T‐ARMS‐PCR and PCR‐RFLP methods. Genotype and allele distributions were compared between groups, Hardy–Weinberg equilibrium was assessed in controls, and crude odds ratios (ORs) were calculated under different genetic models. Multivariable logistic regression was also performed to estimate age‐ and sex‐adjusted ORs. Bonferroni correction was applied to account for multiple comparisons.

**Results:**

Significant associations with increased T2DM risk were observed for miR‐499 rs3746444 C allele (OR = 1.79, 95% CI = 1.34–2.39; *p* < 0.001), miR‐27a rs895819 G allele (OR = 1.89, 95% CI = 1.43–2.51; *p* < 0.001), and miR‐34a rs6577555 A allele (OR = 2.91, 95% CI = 2.19–3.88; *p* < 0.001). These associations remained evident in age‐ and sex‐adjusted codominant logistic regression models. In contrast, miR‐124a rs531564 and miR‐146a rs2910164 were not significantly associated with T2DM risk.

**Conclusion:**

The findings suggest that miR‐499 rs3746444, miR‐27a rs895819/rs895519, and miR‐34a rs6577555 polymorphisms are associated with T2DM susceptibility in this Iranian population. However, these results indicate association rather than causation, and further large‐scale, multiethnic, and functional studies are needed to confirm their biological and clinical relevance.

## 1. Introduction

Type 2 diabetes is a noncommunicable disease and a global challenge characterized by insulin resistance and decreased pancreatic beta‐cell capacity to produce insulin [1]. The long‐term complications of this disease are classified into microvascular (nephropathy, retinopathy, and neuropathy) and macrovascular (cardiovascular diseases, stroke, and diabetic foot) complications [2].

The projected prevalence of this disease was 108 million in 1980, 422 million in 2014, 451 million in 2017, and by 2045, the global population of affected individuals is anticipated to reach 693 million [3]. Managing this illness profoundly affects the patient′s life, family, and the national healthcare infrastructure [2, 4].

The likelihood of developing Type 2 diabetes is influenced by environmental factors, such as rapid economic development, urbanization, sedentary lifestyles and unhealthy dietary patterns [5], and genetic factors [6]. Among the genetic factors, microRNAs are increasingly recognized for their roles in regulating gene expression at the posttranscriptional level [7]. These small, noncoding RNAs (approximately 22 nucleotides in length) can either activate (by interacting with gene promoters) or repress gene expression (by modulating translation or facilitating mRNA degradation) [8]. They are involved in various cellular pathways, including cell growth [9], differentiation [10], development [11], apoptosis [12], and inflammation [13]. Examples of these microRNAs include miR‐499, miR‐27a, miR‐34a, miR‐124a, and miR‐146a. Because of their role in regulating inflammation (miR‐499a, miR‐146a) [14, 15], insulin signaling and beta‐cell function (miR‐27a, miR‐34a, miR‐124a, miR‐146a, and miR‐499a) [16–18], these microRNAs have been investigated in metabolic disorders, including Type 2 diabetes. However, most available evidence relates to miRNA expression and biological activity rather than to the specific polymorphisms analyzed in the present study [19, 20].

Several studies have shown that genetic polymorphisms in microRNAs, such as miR‐499 (rs3746444), miR‐27a (rs895519), miR‐34a (rs6577555), miR‐146a (rs2910164), and miR‐124a (rs531564), can affect their normal function and influence susceptibility to various diseases. For instance, miR‐499 (rs3746444) has been linked to glioma in the Chinese population [21], miR‐27a (rs895519) to colorectal cancer risk in the Chinese population [22], miR‐34a (rs6577555) to renal cell carcinoma in the Iranian population [23], miR‐124a (rs531564) to systemic lupus erythematosus and rheumatoid arthritis in the Iranian population [24], and miR‐146a (rs2910164) to acute coronary syndromes in the Chinese population [25].

The connection between these miRNA polymorphisms and Type 2 diabetes is not thoroughly explored and has been the focus of only a limited number of studies, particularly in Iranian populations. This research was conducted to examine the prevalence of specific miRNA polymorphisms among individuals with Type 2 diabetes compared with healthy controls, which is aimed at identifying possible new biomarkers for disease prevention and management.

## 2. Material and Methods

### 2.1. Subjects

This case–control study included 200 patients with Type 2 diabetes mellitus (T2DM) and 202 unrelated healthy controls. Participants were recruited between 2017 and 2019 from Ali Ibn AbiTalib Hospital, Zahedan, Iran. To reduce potential population stratification, cases and controls were recruited from the same geographic region and during the same study period. All participants were Iranian individuals residing in Zahedan and surrounding areas in southeastern Iran.

T2DM was diagnosed according to the American Diabetes Association (ADA) criteria, defined as fasting plasma glucose (FPG) ≥126 mg/dL, or glycated hemoglobin (HbA1c) ≥6.5%, or a documented clinical diagnosis of T2DM by an endocrinologist. All patients had confirmed T2DM at the time of recruitment. Information regarding disease duration, treatment regimens, and diabetes‐related complications was not systematically available and, therefore, was not included in the present analysis. The control group consisted of unrelated individuals without a history of diabetes or other major chronic metabolic disorders. Controls were recruited from individuals undergoing routine health examinations at the same medical center during the same time period. Individuals with self‐reported diabetes, a family history of diabetes in first‐degree relatives, or clinical evidence of metabolic disease were excluded.

Cases and controls were frequency‐matched by age and sex. Continuous variables such as age were comparable between groups (*p* > 0.05). However, detailed information regarding body mass index (BMI), lipid profile, lifestyle factors (e.g., smoking and physical activity), and medication use was not available for all participants and, therefore, could not be incorporated into the analysis. All participants were of Iranian origin and residents of Zahedan and surrounding areas. Although the study population was geographically restricted to southeastern Iran, detailed ancestry‐informative markers were not assessed. Therefore, the possibility of residual population stratification cannot be completely excluded and should be considered when interpreting the findings.

Written informed consent was obtained from all participants prior to enrollment, and the study protocol was approved by the ethics committee of National Institute for Medical Research Development (Code No. IR.NIMAD.REC.1396.251), in accordance with the Declaration of Helsinki.

### 2.2. Genotyping

The genomic DNA samples were extracted using the salting‐out method and were collected in separate special tubes containing EDTA.

The primers used for detection of miRs polymorphisms are shown in Table 1. Genotyping of miR‐27a rs895519 and miR‐146a rs2910164 was performed using T‐ARMS‐PCR assay, following protocols previously reported in the literature [26]. For miR‐499 rs3746444, miR‐34a rs6577555, and miR‐124a rs531564, the PCR‐RFLP technique was utilized. The PCR amplification was performed using Prime Taq premix (Genetbio, South Korea) in accordance with the manufacturer′s instructions. Each 0.20 mL PCR reaction tube contained approximately 1 *μ*L of genomic DNA (~100 ng/ml), 1 *μ*L of each primer (10 *μ*M), 10 *μ*Lof 2X Prime Taq Premix, and an appropriate volume of ddH_2_O.

**Table 1:** The primers used for miRs polymorphisms genotyping.

Polymorphism	Sequence (5 ^′^– > 3 ^′^)	Restriction enzyme	Product size (bp)
miR‐499 rs3746444	F: CAAAGTCTTCACTTCCCTGCCAR: GATGTTTAACTCCTCTCCACGTGATC	BclI	C = 146T = 122 + 24
miR‐27a rs895519	FO: GCTTGACCCCTGTTCCTGCTGAACTGA	—	Control = 352T = 225C = 184b
	RO: TTGCTTCCTGTCACAAATCACATTGCCA		
	FI: GGAACTTAGCCACTGTGAACACGACTTTGC		
	RI: CTTAGCTGCTTGTGAGCAGGGTCCCCA		
miR‐34a rs6577555	F: CAGCCTGGTTAACATAGCCAGAGC	Eco24I	A = 203C = 180 + 23
	R: CATGCTGACTTTTCAACCACTGTC		
miR‐124a 531564	F: TACAATTAAGGCACGCGGTGA	Alw26I	C = 410G = 234 + 176
	R: ACGGAGGAAGGTGTTGACCC		
miR‐146a 2910164	FO: GGCCTGGTCTCCTCCAGATGTTTAT	—	Control = 364G = 249C = 169
	RO: ATACCTTCAGAGCCTGAGACTCTGCC		
	FI (C allele): ATGGGTTGTGTCAGTGTCAGACGTC		
	RI (G allele): GATATCCCAGCTGAAGAACTGAATTTGAC		

The thermal cycling parameters were as follows: an initial denaturation step at 95°C for 5 min, then 30 cycles of 30 s at 95°C, 30 s at the annealing temperature specific to each polymorphism (64°C for rs3746444, 68°C for rs895519, 62°C for rs6577555, 67°C for rs531564, and 62°C for rs2910164), and 30 s at 72°C, concluding with a final extension step at 72°C for 10 min. For the detection of rs3746444, rs6577555, and rs531564 polymorphisms, 10 *μ*L of the PCR product was subjected to restriction enzyme digestion as indicated in Table 1. All amplified PCR products were separated using 2% agarose gel electrophoresis containing 0.5 *μ*g/mL ethidium bromide and visualized under UV illumination.

To ensure genotyping accuracy, approximately 10% of the samples were randomly selected and regenotyped independently. The concordance rate between initial and repeated genotyping was 100%, indicating high reproducibility. All genotyping procedures were performed blinded to case‐control status to minimize potential bias. Negative controls (no‐template controls) were included in each PCR run to monitor contamination.

### 2.3. Data Analysis

Statistical analyses were performed using SPSS software Version 22. Continuous variables, including age, were expressed as mean ± standard deviation (SD) and compared between groups using Student′s *t*‐test. Categorical variables, including sex distribution, genotype frequencies, and allele frequencies, were analyzed using the chi‐square test or Fisher′s exact test when appropriate. Odds ratios (ORs) and 95% confidence intervals (CIs) were calculated using crude logistic regression under codominant, dominant, recessive, and allelic genetic models to evaluate the association between each polymorphism and T2DM risk.

In addition to crude logistic regression, multivariable logistic regression analyses were performed to estimate adjusted odds ratios (aORs) and 95% CIs for the association between each miRNA polymorphism and T2DM risk under the codominant model. Age and sex were included as covariates in the adjusted models. Each single nucleotide polymorphism (SNP) was analyzed in a separate logistic regression model to avoid overfitting and to evaluate the independent association of each polymorphism with T2DM susceptibility.

Hardy–Weinberg equilibrium (HWE) was assessed in the control group using the chi‐square goodness‐of‐fit test. To account for multiple comparisons, Bonferroni correction was applied separately for each SNP across the tested genetic models. Because five comparisons were evaluated per SNP, the adjusted significance threshold was set at *α* = 0.05/5 = 0.01. Bonferroni‐adjusted *p* values were calculated by multiplying the original *p* values by five, with values greater than 1.00 reported as 1.00. Results with adjusted *p* values<0.01 were considered statistically significant after correction.

Statistical power was estimated based on the observed genotype distributions, case‐control sample size, and detected effect sizes. These calculations were used to assess whether the study had adequate sensitivity to detect the observed associations, particularly for genotypes with low frequency. A two‐sided *p* value < 0.05 was considered statistically significant unless otherwise specified.

## 3. Results

A total of 402 individuals participated in this case–control study. The case group comprised 200 patients with T2DM, including 41 men and 159 women, with a mean age of 56.00 ± 8.78 years. The control group included 202 healthy individuals, including 58 men and 144 women, with a mean age of 55.16 ± 9.96 years. No significant differences in age or sex distribution were observed between the two groups (*p* = 0.370 and *p* = 0.064, respectively).

In the control group, the observed variant allele frequencies were 30.7% for miR‐499 rs3746444 C, 36.9% for miR‐27a rs895819 G, 36.9% for miR‐34a rs6577555 A, 11.4% for miR‐124a rs531564 G, and 37.6% for miR‐146a rs2910164 C. These frequencies were compared with publicly available and previously reported population data, as summarized in Table 2. Overall, the observed allele frequencies were compatible with common population variation, although some differences were noted compared with global, European, or East Asian reference data.

**Table 2:** Comparison of allele frequencies observed in the control group with publicly available or previously reported population data.

miRNA polymorphism	Allele compared	Present study controls *n*/*N*	Present study allele frequency	Public/literature reference frequency ^∗^	Reference population/source
miR‐499 rs3746444	C allele ^∗^	124/404	0.307	0.184	1000 Genomes–based miRNA variant analysis [27]
miR‐27a rs895819	G allele	149/404	0.369	0.360	Reported MAF in a published pharmacogenetic cohort [28]
miR‐34a rs6577555	A allele	149/404	0.369	0.202	Chinese control population [29]
miR‐124a rs531564	G allele	46/404	0.114	0.178	Chinese database/reference frequency [30]
miR‐146a rs2910164	C allele	152/404	0.376	0.22–0.26 in Europeans; approximately 0.30 to 0.40 in Han Chinese	Ensembl/Han Chinese and European population comparison reported by Li et al. [30]

*Note:* Public or literature reference frequencies were obtained from previously reported population datasets or large‐scale public variant resources cited in the table.  ^∗^rs3746444 is commonly reported as A>G for miR‐499a in many databases/publications, whereas the present PCR‐RFLP assay reports the opposite strand as T>C; therefore, allele orientation should be harmonized carefully when comparing databases.

Abbreviation: MAF, minor allele frequency.

The genotype distributions of all five polymorphisms were consistent with HWE in the control group (*p* > 0.05 for all), indicating no evidence of genotyping error or population stratification. The frequencies of genotypes and alleles of the miR‐499, miR‐27a, miR‐34a, miR‐124a, and miR‐146a gene polymorphisms in cases and controls are summarized in Table 3.

**Table 3:** Association between miRNA gene polymorphisms and risk of Type 2 diabetes mellitus.

miRNA (rs ID)	Model	Genotype/allele	Control *n* (%)	Case *n* (%)	OR (95% CI)	*p*	Bonf^#^	HWE ^∗^
miR‐499 (rs3746444)	Codominant	T/T	102 (50.5%)	62 (31.0%)	Reference	—	—	0.42
		T/C	76 (37.6%)	99 (49.5%)	2.14 (1.39–3.31)	0.001	0.005	
		C/C	24 (11.9%)	39 (19.5%)	2.67 (1.47–4.86)	0.001	0.005	
	Dominant	T/C+C/C	100 (49.5%)	108 (69.0%)	1.78 (1.17–2.69)	0.009	0.045	
	Recessive	C/C	24 (11.9%)	39 (19.5%)	1.80 (1.04–3.12)	0.049	0.245	
	Allele	T/C	280/124	223/177	1.79 (1.34–2.39)	< 0.001	< 0.005	

miR‐27a (rs895519)	Codominant	A/A	77 (38.1%)	39 (19.5%)	Reference	—	—	0.19
		A/G	101 (50.0%)	112 (56.0%)	2.19 (1.37–3.50)	0.001	0.005	
		G/G	24 (11.9%)	49 (24.5%)	4.03 (2.16–7.51)	< 0.001	< 0.005	
	Dominant	A/G+G/G	125 (61.9%)	161 (80.5%)	2.54 (1.62–3.99)	< 0.001	< 0.005	
	Recessive	G/G	24 (11.9%)	49 (24.5%)	2.41 (1.41–4.11)	0.002	0.010	
	Allele	A/G	255/149	190/210	1.89 (1.43–2.51)	< 0.001	< 0.005	

miR‐34a (rs6577555)	Codominant	C/C	76 (37.6%)	24 (12.0%)	Reference	—	—	0.26
		C/A	103 (51.0%)	100 (50.0%)	3.07 (1.80–5.25)	< 0.001	< 0.005	
		A/A	23 (11.4%)	76 (38.0%)	10.46 (5.44–20.13)	< 0.001	< 0.005	
	Dominant	C/A+A/A	126 (62.4%)	176 (88.0%)	4.42 (2.65–7.39)	< 0.001	< 0.005	
	Recessive	A/A	23 (11.4%)	76 (38.0%)	4.77 (2.84–8.02)	< 0.001	< 0.005	
	Allele	C/A	255/149	148/252	2.91 (2.19–3.88)	< 0.001	< 0.005	

miR‐124a (rs531564)	Codominant	C/C	160 (79.2%)	157 (78.5%)	Reference	—	—	0.75
		C/G	38 (18.8%)	41 (20.5%)	1.10 (0.67–1.80)	0.706	1.000	
		G/G	4 (2.0%)	2 (1.0%)	0.51 (0.09–2.82)	0.440	1.000	
	Dominant	C/G+G/G	42 (20.8%)	43 (21.5%)	1.04 (0.65–1.68)	0.959	1.000	
	Recessive	G/G	4 (2.0%)	2 (1.0%)	0.50 (0.09–2.76)	0.690	1.000	
	Allele	C/G	358/46	355/45	0.99 (0.64–1.53)	1.000	1.000	

miR‐146a (rs2910164)	Codominant	G/G	76 (37.6%)	81 (40.5%)	Reference	—	—	0.31
		G/C	100 (49.5%)	99 (49.5%)	0.93 (0.61–1.41)	0.730	1.000	
		C/C	26 (12.9%)	20 (10.0%)	0.72 (0.37–1.40)	0.334	1.000	
	Dominant	G/C+C/C	126 (62.4%)	119 (59.5%)	0.89 (0.59–1.32)	0.626	1.000	
	Recessive	C/C	26 (12.9%)	20 (10.0%)	0.75 (0.41–1.40)	0.455	1.000	
	Allele	G/C	252/152	261/139	0.88 (0.66–1.18)	0.439	1.000	

Abbreviations: CI, confidence interval; OR, odds ratio; SNP, single nucleotide polymorphism; T2DM, Type 2 diabetes mellitus.

^∗^HWE: Hardy–Weinberg equilibrium assessed in the control group using the chi‐square test. HWE p values > 0.05 indicate no deviation from equilibrium.

#Bonf: Bonferroni correction applied on a per‐SNP basis to account for multiple testing (adjusted significance threshold *α* = 0.05/5 = 0.01). *p* values below 0.01 were considered statistically significant after correction.

For miR‐499 rs3746444, the TT, TC, and CC genotypes were observed in 62/200, 99/200, and 39/200 patients with T2DM, respectively, compared with 102/202, 76/202, and 24/202 controls. For miR‐27a rs895519, the AA, AG, and GG genotypes were observed in 39/200, 112/200, and 49/200 cases, respectively, compared with 77/202, 101/202, and 24/202 controls. For miR‐34a rs6577555, the CC, CA, and AA genotypes were observed in 24/200, 100/200, and 76/200 cases, respectively, compared with 76/202, 103/202, and 23/202 controls. Moreover, for miR‐124a rs531564, the CC, CG, and GG genotypes were observed in 157/200, 41/200, and 2/200 cases, respectively, compared with 160/202, 38/202, and 4/202 controls. For miR‐146a rs2910164, the GG, GC, and CC genotypes were observed in 81/200, 99/200, and 20/200 cases, respectively, compared with 76/202, 100/202, and 26/202 controls.

The data demonstrated that the rs3746444 polymorphism significantly associated with increased risk of developing T2DM in various genetic models, including codominant (OR = 2.14, 95% CI = 1.39–3.31; *p* = 0.001 for TC vs. TT and OR = 2.67, 95% CI = 1.47–4.86; *p* = 0.001 for CC vs. TT), dominant (OR = 1.78, 95% CI = 1.17–2.69; *p* = 0.009 for TC + CC vs. TT), recessive (OR = 1.80, 95% CI = 1.04–3.12; *p* = 0.049 for CC vs. TT+TC), and allelic (OR = 1.79, 95% CI = 1.34–2.39; *p* < 0.001 for C vs. T).

Regarding miR‐27a polymorphism, our analysis showed that rs895519 was significantly associated with an increased risk of Type 2 diabetes in codominant (OR = 2.19, 95% CI = 1.37–3.50; *p* = 0.001 for AG vs. AA and OR = 4.03, 95% CI = 2.16–7.51; *p* < 0.001 for GG vs. AA), dominant (OR = 2.54, 95% CI = 1.62–3.99; *p* < 0.001 for AG+GG vs. AA), recessive (OR = 2.41, 95% CI = 1.41–4.11; *p* = 0.002 for GG vs. AA+AG), and allelic (OR = 1.89, 95% CI = 1.43–2.51; *p* < 0.001 for G vs. A) models.

For miR‐34a polymorphism, rs6577555 was found to be significantly correlated with elevation risk of Type 2 diabetes in codominant (OR = 3.07, 95% CI = 1.80–5.25; *p* < 0.001 for CA vs. CC and OR = 10.46, 95% CI = 5.44–20.13; *p* < 0.001 for AA vs. CC), dominant (OR = 4.42, 95% CI = 2.65–7.39; *p* < 0.001 for CA+AA vs. CC), recessive (OR = 4.77, 95% CI = 2.84–8.02; *p* <0.001 for AA vs CC + CA), and allelic (OR = 2.91, 95% CI = 2.19–3.88; *p* < 0.001 for A vs. C) models. However, no significant associations were found between the miR‐124a rs531564 or miR‐146a rs2910164 polymorphisms and the risk of Type 2 diabetes in our study.

To account for multiple comparisons, Bonferroni correction was applied separately for each SNP across the tested genetic models. Because five comparisons were evaluated per SNP, the adjusted significance threshold was set at *α* = 0.05/5 = 0.01. Bonferroni‐adjusted *p* values were calculated by multiplying the original *p* values by five, with values greater than 1.00 reported as 1.00. Results with adjusted *p* values < 0.01 were considered statistically significant after correction.

After applying per‐SNP Bonferroni correction (adjusted significance threshold *α* = 0.01), the associations for miR‐34a (rs6577555) remained statistically significant under all genetic models (all adjusted *p* values < 0.005). Similarly, miR‐27a (rs895519) showed significant associations in codominant, dominant, recessive, and allelic models after correction (adjusted *p* ≤ 0.010). For miR‐499 (rs3746444), the codominant and allelic models remained significant following Bonferroni adjustment (adjusted *p* = 0.005 and < 0.005, respectively), whereas the dominant and recessive models were no longer statistically significant after correction. No significant associations were observed for miR‐124a (rs531564) or miR‐146a (rs2910164) under any genetic model after Bonferroni correction.

Power analysis indicated that the study had adequate power to detect large genotype‐specific effects, particularly for miR‐34a rs6577555 AA, whereas power was more limited for modest effects and rare genotype comparisons. Therefore, nonsignificant findings for miR‐124a rs531564 and miR‐146a rs2910164 should be interpreted with caution.

Age‐ and sex‐adjusted logistic regression analyses were performed under the codominant model for each polymorphism, as shown in Table 4. After adjustment for age and sex, miR‐499 rs3746444 remained significantly associated with T2DM risk for both the TC genotype (aOR = 2.187, 95% CI = 1.407–3.401; *p* = 0.001) and CC genotype (aOR = 2.599, 95% CI = 1.424–4.746; *p* = 0.002) compared with the TT genotype. Similarly, miR‐27a rs895819 remained significantly associated with increased T2DM risk for the AG genotype (aOR = 2.189, 95% CI = 1.365–3.511; *p* = 0.001) and GG genotype (aOR = 4.045, 95% CI = 2.160–7.573; *p* < 0.001) compared with the AA genotype. For miR‐34a rs6577555, the CA genotype (aOR = 3.103, 95% CI = 1.807–5.328; *p* < 0.001) and AA genotype (aOR = 10.810, 95% CI = 5.580–20.944; *p* < 0.001) were also significantly associated with increased T2DM risk compared with the CC genotype. In contrast, no significant adjusted associations were observed for miR‐124a rs531564 or miR‐146a rs2910164.

**Table 4:** Age‐ and sex‐adjusted logistic regression analysis of miRNA polymorphisms and Type 2 diabetes mellitus risk.

Polymorphism	Genotype	Cases *n* (%)	Controls *n* (%)	Crude OR (95% CI)	Crude *p*	Adjusted OR (95% CI)	Adjusted *p*
miR‐499 rs3746444	TT	62 (31.0)	102 (50.5)	Reference	—	Reference	—
	TC	99 (49.5)	76 (37.6)	2.14 (1.39–3.31)	0.001	2.187 (1.407–3.401)	0.001
	CC	39 (19.5)	24 (11.9)	2.67 (1.47–4.86)	0.001	2.599 (1.424–4.746)	0.002
miR‐27a rs895519	AA	39 (19.5)	77 (38.1)	Reference	—	Reference	—
	AG	112 (56.0)	101 (50.0)	2.19 (1.37–3.50)	0.001	2.189 (1.365–3.511)	0.001
	GG	49 (24.5)	24 (11.9)	4.03 (2.16–7.51)	< 0.001	4.045 (2.160–7.573)	< 0.001
miR‐34a rs6577555	CC	24 (12.0)	76 (37.6)	Reference	—	Reference	—
	CA	100 (50.0)	103 (51.0)	3.07 (1.80–5.25)	< 0.001	3.103 (1.807–5.328)	< 0.001
	AA	76 (38.0)	23 (11.4)	10.46 (5.44–20.13)	< 0.001	10.810 (5.580–20.944)	< 0.001
miR‐124a rs531564	CC	157 (78.5)	160 (79.2)	Reference	—	Reference	—
	CG	41 (20.5)	38 (18.8)	1.10 (0.67–1.80)	0.706	1.076 (0.655–1.769)	0.771
	GG	2 (1.0)	4 (2.0)	0.51 (0.09–2.82)	0.440	0.463 (0.082–2.605)	0.382
miR‐146a rs2910164	GG	81 (40.5)	76 (37.6)	Reference	—	Reference	—
	GC	99 (49.5)	100 (49.5)	0.93 (0.61–1.41)	0.730	0.918 (0.603–1.399)	0.692
	CC	20 (10.0)	26 (12.9)	0.72 (0.37–1.40)	0.334	0.705 (0.361–1.375)	0.305

*Note:* Adjusted ORs were calculated using multivariable logistic regression adjusted for age and sex under the codominant model. Each SNP was analyzed in a separate model.

Abbreviations: OR, odds ratio; CI, confidence interval.

## 4. Discussion

T2DM is one of the most common metabolic diseases worldwide, mainly caused by the dual issues of impaired insulin production by pancreatic cells and a diminished response to insulin by target tissues. The interplay between these two key elements serves as a major catalyst in the development of T2DM. Beyond the well‐known environmental risk factors, such as obesity, age, and dietary habits, genetic factors are also considered to be associated with susceptibility to T2DM.

MicroRNAs, which are one of the genetic factors, are RNAs that vary in length from approximately 18 to 24 nucleotides. MicroRNAs are a class of short noncoding RNAs that significantly contribute to the posttranscriptional regulation of gene expression, as well as the regulation of cellular metabolic pathways and maturation processes [27]. In this study, we focused on five miRNAs: miR‐499, miR‐27a, miR‐34a, miR‐124a, and miR‐146a. These miRNAs have been implicated as potential biomarkers for T2DM because of their involvement in inflammation regulation (miR‐499a and miR‐146a) as well as in insulin signaling and pancreatic beta‐cell functionality (miR‐27a, miR‐34a, miR‐124a, miR‐146a, and miR‐499a) [4, 28]. However, the present study evaluated only genetic polymorphisms and did not assess miRNA expression levels or functional consequences.

Additionally, polymorphisms in miRNA genes may be associated with altered regulation of target genes and variation in disease susceptibility, although their functional effects require further validation [19, 29]. This study is aimed at evaluating the association of miR‐27a (rs895519), miR‐499 (rs3746444), miR‐34a (rs6577555), miR‐124a (rs531564), and miR‐146a (rs2910164) polymorphisms with T2DM in an Iranian population.

Comparison of allele frequencies with public and previously reported population data provides additional context for interpreting the present findings. The miR‐27a rs895819/rs895519 G allele frequency in our controls was very close to the reported literature MAF of approximately 0.36, whereas the miR‐499 rs3746444 variant allele frequency was higher than the 1000 Genomes–based MAF reported in a previous miRNA‐variant analysis. The miR‐34a rs6577555 A allele frequency was also higher in our controls than that reported in a Chinese control population. For miR‐146a rs2910164, previous data indicate substantial population differences, with the C allele reported at approximately 22%–26% in European populations and the G allele being the minor allele at approximately 30%–40% in Han Chinese populations [19, 30–32].

The results of our study revealed that the miR‐499 (rs3746444), miR‐27a (rs895519), and miR‐34a (rs6577555) gene polymorphisms were significantly associated with increased susceptibility to T2DM. Analysis of SNPs under different genetic models (codominant, dominant, recessive, and allelic) showed that individuals carrying specific alleles, such as the C allele in rs3746444, the G allele in rs895519, and the A allele in rs6577555, demonstrated higher odds of T2DM compared with noncarriers.

On the other hand, no significant connection was identified between the miR‐124a (rs531564) and miR‐146a (rs2910164) polymorphisms and T2DM. However, the absence of statistical significance does not exclude the possibility of modest effects, and the study may have been underpowered to detect small associations. Larger studies are required to clarify their potential contribution.

Interestingly, our findings regarding miR‐34a (rs6577555) differ from a study conducted by Sun et al. [33] in the Chinese population, which reported no link between this polymorphism and T2DM. In contrast, our study found a strong association between rs6577555 (C>A) and increased diabetes risk. Similarly, Lee et al. [34] observed the same polymorphism to be associated with recurrent implantation failure, consistent with our observations. Previous experimental studies have suggested that miR‐34a may influence pancreatic *β*‐cell apoptosis (e.g., via Bcl‐2 inhibition) and insulin secretion (via VAMP2 regulation) [35]. Importantly, these studies evaluated miR‐34a expression or functional activity and did not specifically assess the rs6577555 polymorphism.

However, the relatively large effect estimate observed for the miR‐34a rs6577555 AA genotype should be interpreted with caution. T2DM is a complex polygenic and multifactorial disease, and individual common variants usually exert modest effects. Therefore, the high OR observed in this study may reflect population‐specific genetic structure, the relatively small number of individuals with some genotypes, unmeasured confounding, or possible effect‐size inflation commonly observed in moderate‐sized candidate‐gene studies. Although the association remained significant after adjustment for age and sex, replication in independent cohorts and functional validation are required before this variant can be considered a robust risk marker.

Our results demonstrated that the miR‐499 (rs3746444 T>C) polymorphism was associated with T2DM susceptibility. Although previous association studies have linked this polymorphism to various diseases [36, 37], many of these investigations were conducted in cancer‐related settings [38]. Such findings cannot be directly extrapolated to T2DM pathogenesis.

Experimental studies have shown that miR‐499a is involved in cellular senescence, inflammation, apoptosis, and immune responses, and may influence insulin signaling pathways through PTEN regulation [39]. Nevertheless, these studies primarily address miRNA‐level biological functions and do not demonstrate that the rs3746444 variant alters miR‐499a expression, maturation, or target binding in the context of T2DM. The functional consequences of this polymorphism therefore remain to be determined.

Additionally, our analysis indicated that the miR‐27a (rs895519A>G) variant was associated with increased odds of T2DM. Some previous association studies of this polymorphism were conducted in cancer populations [40], and although they demonstrate that rs895519 may influence disease susceptibility in certain contexts, they do not provide direct evidence regarding its role in metabolic disorders. Although miR‐27a has been implicated in insulin resistance and glucose metabolism through the PI3K/Akt pathway, these findings are based on studies evaluating miR‐27a expression or functional activity rather than the rs895519 polymorphism itself. The specific biological effect of this variant on miRNA processing or target regulation was not assessed in the present study.

Our results showed no association between miR‐124a (rs531564G>C) and miR‐146a (rs2910164G>C) variants and T2DM occurrence, consistent with findings reported by Wang et al. [41] and Cheng et al. [42]. Although rs2910164 has been associated with other diseases, including cancer [43, 44], such findings should not be interpreted as evidence of functional relevance for T2DM.

From a biological perspective, one plausible hypothesis is that genetic variation within pre‐miRNA regions may subtly influence miRNA maturation efficiency, expression levels, or regulatory interactions with target genes, thereby contributing to interindividual differences in susceptibility to metabolic disorders; however, this possibility remains speculative and requires direct functional validation in T2DM‐specific experimental models.

Importantly, given the observational case–control design, the lack of longitudinal data, and the absence of functional validation, the findings of this study should be interpreted as associations rather than causal relationships. The sample size may limit statistical power for detecting modest effects. Furthermore, this study was conducted in a geographically restricted Iranian population, and the findings may not be generalizable to other ethnic groups. Potential environmental and metabolic confounders (e.g., BMI, lipid profile, medication use, physical activity, and socioeconomic status) were not comprehensively assessed. Epigenetic modifications and gene–environment interactions were also not evaluated. Moreover, in silico prediction of the structural or regulatory consequences of the investigated polymorphisms was not performed in the present study. Future studies should incorporate computational prediction and experimental validation to determine whether these variants alter miRNA maturation, expression, or target‐gene regulation.

Future research should include larger multicenter cohorts, functional analyses of polymorphism effects on miRNA expression and target regulation, and longitudinal designs to evaluate temporal relationships. Integration of environmental exposures and advanced genomic approaches such as GWAS may further clarify the complex genetic architecture of T2DM.

## 5. Conclusion

This study identified significant associations between miR‐499 (rs3746444), miR‐27a (rs895519), and miR‐34a (rs6577555) polymorphisms and T2DM susceptibility in an Iranian population. These findings contribute to the growing body of evidence supporting the involvement of miRNA‐related genetic variation in metabolic disorders. However, the present results demonstrate association rather than causation. No significant associations were observed for miR‐124a rs531564 and miR‐146a rs2910164 variants, although larger studies are required to exclude modest effects. Although these miRNAs are implicated in insulin signaling, inflammation, and *β*‐cell regulation based on previous experimental studies, the functional consequences of the investigated polymorphisms were not examined in the current study. Therefore, these variants should be considered genetic factors associated with susceptibility rather than validated biomarkers or predictive tools. Further large‐scale, multiethnic, and functional investigations are necessary to confirm these findings and determine their potential clinical relevance.

## Author Contributions

M.H. and G.B. conceived and designed the study. M.B. (Mehrdad Behdani), M.B.(Masoumeh Bahari), and M.T. performed the experiments and analyzed the data. M.A.K. performed data collection. All authors contributed to the writing of the manuscript.

## Funding

This study was funded by the National Institute for Medical Research Development (No.963492).

## Disclosure

All authors reviewed and approved the final manuscript.

## Ethics Statement

This study was approved by the ethics committee of National Institute for Medical Research Development (Code No. IR.NIMAD.REC.1396.251). Written informed consent was obtained from all participants prior to inclusion.

## Conflicts of Interest

The authors declare no conflicts of interest.

## Data Availability

The data that support the findings of this study are available on request from the corresponding author. The data are not publicly available due to privacy or ethical restrictions.
